# Identification of Novel Serodiagnostic Signatures of Typhoid Fever Using a *Salmonella* Proteome Array

**DOI:** 10.3389/fmicb.2017.01794

**Published:** 2017-09-19

**Authors:** Thomas C. Darton, Stephen Baker, Arlo Randall, Sabina Dongol, Abhilasha Karkey, Merryn Voysey, Michael J. Carter, Claire Jones, Krista Trappl, Jozelyn Pablo, Chris Hung, Andy Teng, Adam Shandling, Tim Le, Cassidy Walker, Douglas Molina, Jason Andrews, Amit Arjyal, Buddha Basnyat, Andrew J. Pollard, Christoph J. Blohmke

**Affiliations:** ^1^Oxford Vaccine Group, Centre for Clinical Vaccinology and Tropical Medicine, Department of Paediatrics, and the Oxford National Institutes for Health Research Biomedical Research Centre, University of Oxford Oxford, United Kingdom; ^2^The Hospital for Tropical Diseases, Wellcome Trust Major Overseas Programme, Oxford University Clinical Research Unit Ho Chi Minh City, Vietnam; ^3^Department of Infection, Immunity and Cardiovascular Disease, The University of Sheffield Sheffield, United Kingdom; ^4^Antigen Discovery Incorporated, Irvine CA, United States; ^5^Oxford University Clinical Research Unit, Patan Academy of Health Sciences Kathmandu, Nepal; ^6^Nuffield Department of Primary Care Health Sciences, University of Oxford Oxford, United Kingdom; ^7^Division of Infectious Diseases and Geographic Medicine, Stanford University, Stanford CA, United States

**Keywords:** *Salmonella* Typhi, serodiagnostics, antibody response, fever diagnostics, enteric fever, machine learning, controlled human infection model, rapid diagnostic tests

## Abstract

Current diagnostic tests for typhoid fever, the disease caused by *Salmonella* Typhi, are poor. We aimed to identify serodiagnostic signatures of typhoid fever by assessing microarray signals to 4,445 *S*. Typhi antigens in sera from 41 participants challenged with oral *S*. Typhi. We found broad, heterogeneous antibody responses with increasing IgM/IgA signals at diagnosis. In down-selected 250-antigen arrays we validated responses in a second challenge cohort (*n* = 30), and selected diagnostic signatures using machine learning and multivariable modeling. In four models containing responses to antigens including flagellin, OmpA, HlyE, sipC, and LPS, multi-antigen signatures discriminated typhoid (*n* = 100) from other febrile bacteremia (*n* = 52) in Nepal. These models contained combinatorial IgM, IgA, and IgG responses to 5 antigens (ROC AUC, 0.67 and 0.71) or 3 antigens (0.87), although IgA responses to LPS also performed well (0.88). Using a novel systematic approach we have identified and validated optimal serological diagnostic signatures of typhoid fever.

## Introduction

Typhoid fever is a febrile illness common in tropical regions of South and Southeast Asia, and is becoming increasingly recognized in sub-Saharan Africa (Crump et al., 2015; Wain et al., 2015). The causative bacterium *Salmonella enterica* serovar Typhi (*S.* Typhi) is transmitted between humans by the faeco-oral route, and is associated with 12 to 27 million illnesses each year (Crump et al., 2004; Buckle et al., 2012; Mogasale et al., 2014). Estimates of typhoid disease burden are broad and likely inaccurate due to lack of systematic studies and inadequate diagnostic methods (Crump et al., 2008; Crump, 2014; John et al., 2016). Management of individual cases may also be similarly compromised; whereas rapid diagnostic tests (RDTs) have been developed for other common tropical febrile infections, no such tests currently exist for typhoid (Baker et al., 2010; Parry et al., 2011; Andrews and Ryan, 2015).

The diagnosis of typhoid fever is dependent on traditional microbiological techniques and clinical judgment (World Health Organisation, 2003), with blood culture still considered to be the gold-standard. While modern blood culture facilities may achieve a diagnostic sensitivity of 80% and a specificity approaching 100% (Mogasale et al., 2014; Waddington et al., 2014), sensitivity is often compromised due to a low concentration of organisms in the blood on clinical presentation and the use of antimicrobials before hospitalization (Wain et al., 1998; World Health Organisation, 2003). The classic serological method for diagnosing typhoid fever is the Widal test, which measures agglutination of serum antibodies against *S.* Typhi flagellin and lipopolysaccharide (LPS) (Crump et al., 2015). The useful application of the Widal test is complicated in endemic settings, however, due to cross-reactivity with other antigens and the need for either paired samples or population-specific baseline samples (Baker et al., 2010; Keddy et al., 2011). As a result of the low blood volume requirements and possible extrapolation to using non-blood clinical samples, serological responses remain an appealing approach for typhoid diagnostics, although a central shortfall has been a lack of diagnostic antigen candidates for *S.* Typhi (Darton et al., 2014).

We previously established a controlled human infection model (CHIM) of typhoid fever (Waddington et al., 2014; Darton et al., 2016). This model readily lends itself to the interrogation of immune responses after an oral challenge with virulent *S.* Typhi. In tandem, the fabrication of a pan-*Salmonella* proteome array by antigen expression using a coupled *Escherichia coli*-based *in vitro* transcription and translation (IVTT) system has enabled the systematic assessment of humoral antibody responses to vaccination and/or infection (Davies et al., 2005; Liang and Felgner, 2015). Here, we describe an assessment of the humoral immune response after oral challenge with virulent *S.* Typhi, through infection and into convalescence. We aimed to identify and validate novel signatures of antigen/antibody isotype combinations using typhoid CHIMs, before evaluating the performance of these diagnostic signatures in febrile patients in a typhoid-endemic area of Nepal.

## Results

### Discovery of a Diagnostic Signature in a Typhoid CHIM

**FIGURE 1:**
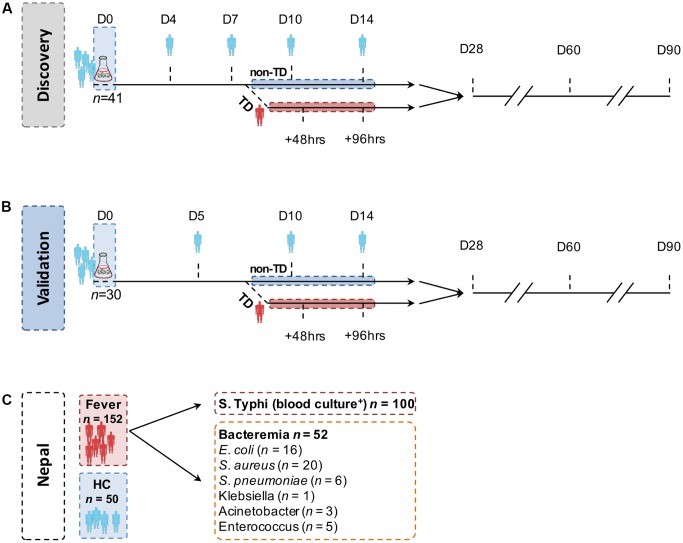
Structure of the controlled human infection models of typhoid fever and endemic cohort. In both **(A)** the discovery set and **(B)** the validation set, study participants ingested 10^3^–10^4^ CFU *Salmonella* Typhi Quailes strain suspended in oral sodium bicarbonate solution on day 0 (D0). Sera samples were collected and probed at the time points indicated. Participants developing an oral temperature ≥38°C sustained for ≥12 h or evidence of bacteremia after challenge were diagnosed with typhoid (TD) and commenced on antimicrobial treatment. All remaining participants not diagnosed during the 14-day period (nTD) were commenced on the same treatment on day 14. **(C)** Samples (serum and blood culture) in the endemic setting cohorts were collected on one occasion at point of hospital presentation. Pathogens isolated from blood cultures collected from other, non-*S.* Typhi bacteraemia cases are listed in the box.

**FIGURE 2:**
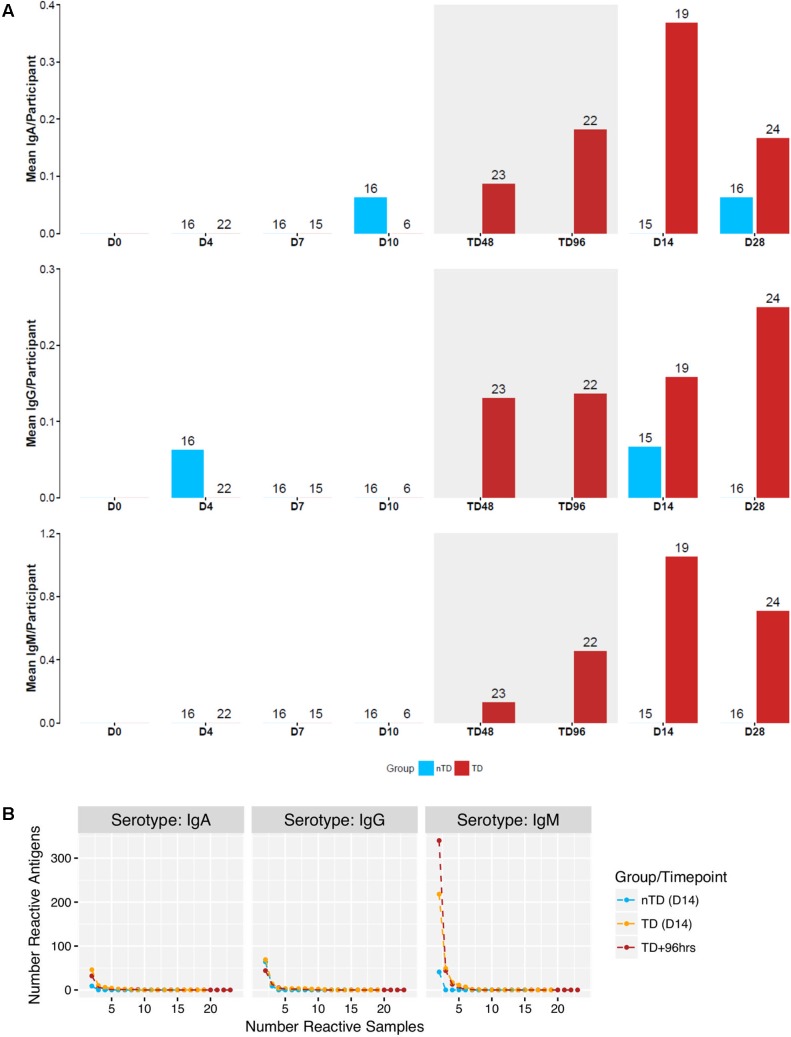
Reactivity of 4445 antigens in samples from a human challenge study performed in Oxford (Discovery set). **(A)** Mean number of creactive antigen per participant at time points D0 – D28 following challenge. Blue: nTD group. Red: TD group. Gray shaded area: acute disease (TD48 and TD96 h). **(B)** Number of reactive antigens in number of samples at TD+96 h, and D14 for both groups.

Arrays consisting of 4,445 *S*. Typhi antigens expressed using IVTT plus purified *S.* Typhi LPS and flagellin were used to probe sera collected from 41 participants challenged with *S*. Typhi (**Figure 1A** and **Table 1**) (Waddington et al., 2014; Darton et al., 2016). We measured IgA, IgM, and IgG reactivity in all individuals up to day 28 after challenge (**Figure 1A** and Supplementary Figure S1). All participants diagnosed with typhoid fever (TD) developed humoral responses; these responses were heterogeneous with few antigens represented across all samples (**Figure 2**). Moreover, TD participants exhibited a broader range of antibody responses for all three isotypes than participants not developing infection (nTD) after challenge.

**Table 1:** Demographics.

Study	Discovery cohort	Validation cohort	Endemic cohort
Identifier	OVG2009/10	OVG2011/02	NA
Location	Oxford, United Kingdom	Oxford, United Kingdom	Kathmandu, Nepal
Source	*S.* Typhi Quailes strain dose-escalation study (10^3^ and 10^4^CFU) (Waddington et al., 2014)	Placebo arm of randomized controlled vaccine/challenge trial (Darton et al., 2016)	Treatment trial and diagnostics sub-study (Arjyal et al., 2016; Darton et al., 2016)
Trial registration	NA	Clinicaltrials.gov (NCT01405521)	Clinicaltrials.gov (NCT01421693)
Sample size, *N*	41	30	202
Confirmed cases^A^, n (% bacteremia)	25 (84)	20 (100)	100 (100)
Exposed, not sick^B^, *n*	16	10	NA
Healthy controls^C^, *n*	41	30	50
Febrile non-typhoid bacteremia, *n*	NA	NA	52^D^
Median age, yrs (interquartile range)	27 (22–37)	23 (21–39)	20 (15–27)^E,F^
Number male (%)	28 (68)	19 (63)	99 (49)^E,G^

*NA, not applicable. CFU, colony-forming units*.

^A^*Case confirmation in the challenge studies was made using clinical (oral temperature ^3^38°C sustained for ^3^12 h continuously) or microbiological (S. Typhi bacteraemia detected in ^3^1 blood culture sample). All confirmed cases in the endemic cohort were confirmed to have bacteremia*.

^B^*CHIM study participants who ingested challenge agent and were monitored for 14 days but did not meet the endpoints for typhoid diagnosis (see A, above)*.

^C^*Healthy controls in the challenge study were paired pre-challenge samples from the same individual*.

^D^*Pathogens isolated (n) were: Staphylococcus aureus (20), Escherichia coli (16), Streptococcus pneumoniae (6), Enterococcus sp. (5), Acinetobacter sp. (3), Streptococcus sp. (1), Klebsiella sp. (1)*.

^E^*Missing data = 6/202 healthy controls*.

^F^*Typhoid patients, 16 (10–20); Healthy controls, 22.5 (19–26.25); Febrile controls, 40 (22–56.75)*.

^G^*Typhoid patients, 67 (67%); Healthy controls, 11 (22%); Febrile controls, 21 (40%)*.

**FIGURE 3:**
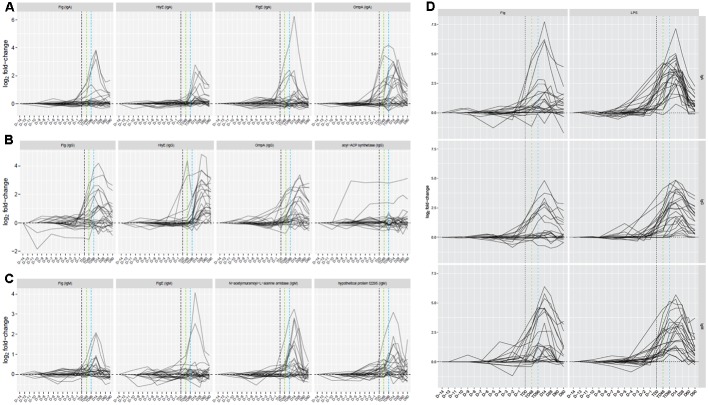
Time course of responses to four selected antigen/antibody isotype combinations by participants challenged and subsequently diagnosed with typhoid fever. **(A)** IgA responses. **(B)** IgG responses. **(C)** IgM responses. **(D)** Responses to purified *S.* Typhi flagellin (0.1 μg) and lipopolysaccharide (LPS, 0.1 μg) as additional antigens included on the array. Vertical black dashed line, TD time point; vertical green dashed line, TD+48hr time point; vertical blue dashed line, TD+96hr time point.

Antigen/isotype combinations reaching predetermined reactivity criteria (fold-change >1.5 from baseline in >2 participants) at day 14 in all participants plus the 96 h after TD (TD96 h) time point in TD participants, according to the outcome after challenge, included flagellar components, HlyE, lipoproteins, regulatory proteins, OmpA and others distributed between all three isotypes (7 IgA, 8 IgG, and 16 IgM antigens; Supplementary Table S1). These data reconfirmed the heterogeneity in responses by TD participants, particularly at early time points (**Figure 3** and Supplementary Figures S2A–C). In contrast, sera from nTD participants exhibited little reactivity to these same antigens (Supplementary Figures S2D–F, S3).

### Validation of Selected Antigen/Antibody Isotypes

Using the discovery set data and previously published data (Lee et al., 2012; Liang et al., 2013; Davies et al., 2016), we produced a down-selected array consisting of 223 *S.* Typhi, 6 dengue virus, and 11 *Plasmodium falciparum* antigens and purified *S.* Typhi antigens, Vi, LPS and flagellin (Supplementary Methods and Table S2). The resulting arrays were probed with sera from an independent cohort of *S*. Typhi challenged participants (validation set, *n* = 30) to verify the results from the discovery cohort (**Figure 1B**) (Darton et al., 2016).

**FIGURE 4:**
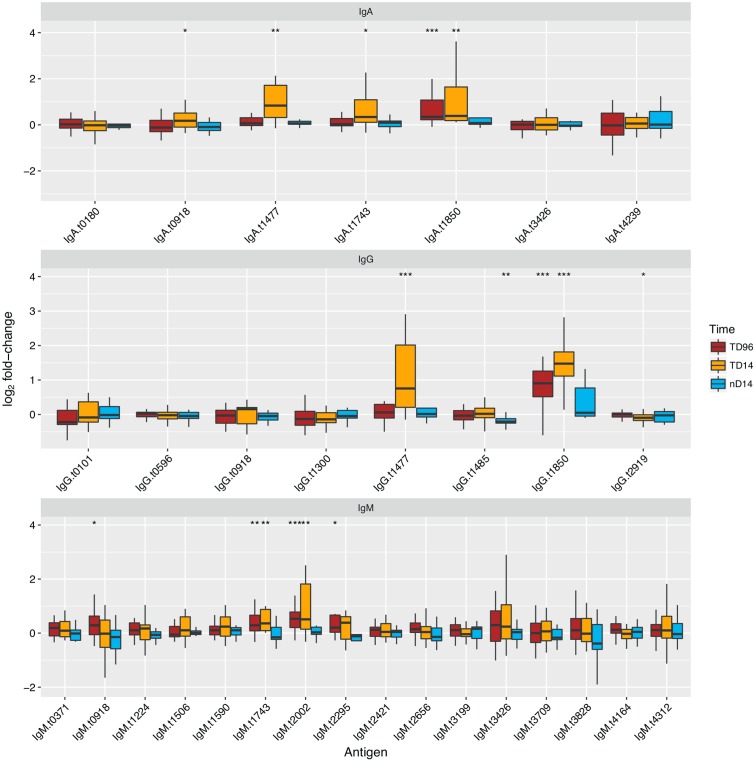
Reactivity and diagnostic performance of antigen/antibody isotype combinations selected from the discovery set and applied to the validation set. Antigen names are given in Supplementary Table S1. Boxplots of fold-change in reactivity between TD+96hr and day 14 time points in TD or day 14 in nTD participants and individual baseline FI values, to antigen/antibody isotype combinations selected from the discovery set. Paired *t*-tests were performed between the time point featured and baseline values. ^∗^*p* < 0.05, ^∗∗^*p* < 0.01, ^∗∗∗^*p* < 0.001.

Of the 31 antigen/antibody isotype combinations selected from the discovery set, six had significant increases in sera reactivity at the TD96 h time point in those diagnosed with typhoid (paired *t*-test) and nine had significant increases in sera reactivity at the day 14 time point (**Figure 4**). We observed significant responses to four antigens per isotype: IgG and IgA with HlyE and OmpA, IgG, and IgM with flagellin and the flagellar hook protein E (FlgE). Unique antigen/antibody isotype signals were observed with IgM against a putative *N*-acetylmuramoyl-L-alanine amidase (t2002) and an uncharacterised protein (t2295), and with IgG against methyl viologen resistance protein SmvA (t1485) and bifunctional protein Aas (t2919).

To further explore the diagnostic potential of the antigen/antibody isotype combinations selected from the discovery set, we plotted receiver operator characteristic (ROC) curves for each combination; we used the fold-change between day 14 and baseline to discriminate all challenge study participants reaching a study diagnosis of typhoid from those who did not (Supplementary Figure S4). These analyses confirmed the discriminative ability of IgA and IgG isotype responses to HlyE (t1477) and OmpA (t1850) based on their high AUC values (>0.75); the IgM responses were less discriminatory.

### Selection of Antigen/Isotype Combination Signatures by Machine Learning

While the initial analysis identified a set of antigens to evaluate in a multivariable model, we further aimed to optimize antigen selection using complementary, data driven approaches to down-select antigen/antibody isotype combinations using machine learning algorithms. To achieve reasonably sized training and test sets and to maximize the available data points included, we used both discovery and validation sets (*n* = 71) and antigen/antibody isotype combinations common to the full and down-selected arrays to form one large data matrix (‘superset’) consisting of 715 features (Supplementary Methods).

A principal component analysis (PCA) of all antigens common to the challenge and Nepal datasets indicated substantial response homogeneity (Supplementary Figure S5); with the exception of two Nepal samples that were excluded from subsequent analyses. We initially tested four different algorithms: partial least squares regression (PLS) (Mevik and Wehrens, 2007), nearest shrunken centroid classification (NSC or PAM) (Tibshirani et al., 2002), radial and linear support vector machines (SVMs) (Karatzoglou et al., 2004).

**FIGURE 5:**
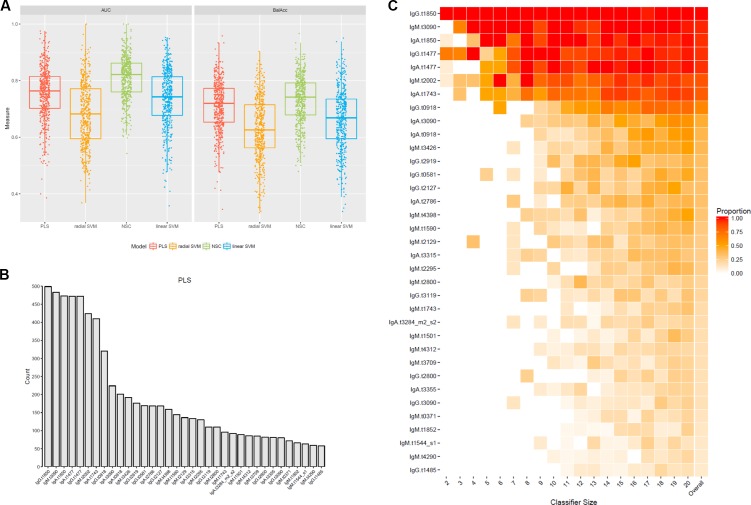
Selection of an antigen/antibody isotype signature using machine learning algorithms applied to the controlled human infection typhoid datasets. **(A)** Test set prediction performance measures AUC receiver operator characteristic (ROC) and balanced accuracies (BalAcc) for four different machine learning models using 500 bootstrap samples of the data. **(B)** Frequency of features selected in each of 500 iterations by the partial least squares (PLS) algorithm. **(C)** Proportions of features selected across all 500 bootstrap samples using the PLS algorithm. Features had to be selected in at least 10% of the bootstrap samples (column ‘overall’). Proportions are split by classifier size. The last column represents the overall proportion across all 500 bootstrap samples.

Overall, radial SVM was the algorithm with the lowest predictive accuracy according to AUROC scores, and the lowest balance accuracy (**Figure 5A**). The NSC method produced high predictive accuracies but only performed well with large classifier sets and thus both methods were excluded from subsequent analyses (**Figure 5A** and Supplementary Figure S6). Due to the performance of the PLS regression and overlap seen with the linear SVM algorithm (Supplementary Figure S7), subsequent analyses were performed using antigens selected by the PLS method only. Mapping the overall selection frequency of specific antigens selected by the PLS model indicated that OmpA (t1850), putative membrane protein (t3090), HlyE (t1477), putative *N*-acetylmuramoyl-L-alanine amidase (t2002) and FlgE (t1743) were selected in >80% of the classifiers built by the PLS algorithm (**Figure 5B**). Using a threshold of >10% selection frequency across all 500 iterations, 35 unique antigen/antibody isotype combinations were selected as candidates for multivariable modeling (**Figure 5C** and Supplementary Table S3).

### Confirmation of Antigen/Antibody Isotype Signature

**FIGURE 6:**
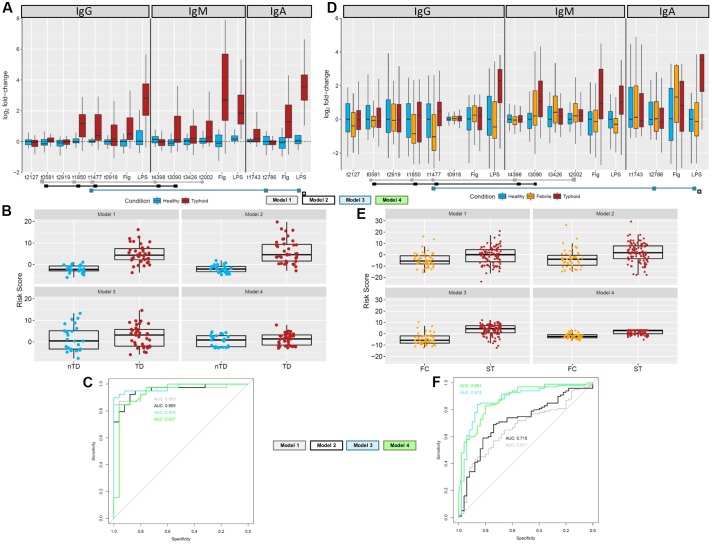
Multivariable analysis to find optimal antigen/antibody isotype signature. **(A)** Fold-change values of 12 target antigens plus flagellin and LPS in the combined Oxford data. Antigens included in one of the final models are indicated by the colored squares below the antigens. **(B)** Risk scores for the Oxford samples based on the antigens and coefficients in the four final models. **(C)** ROC curves for each of the final model based on the Oxford data comparing participants diagnosed with typhoid fever (day 14 or TD96 h; brown) with those who stayed well (day 14; blue). **(D)** Fold-change values of 12 target antigens plus flagellin and LPS in the Nepal data. Fold-changes were generated against the median of the healthy population (*n* = 50). Antigens included in one of the final models are indicated by the colored squares below the antigens. **(E)** Risk scores for the Nepalese febrile control (orange) and typhoid (brown) samples based on the antigens and coefficients in the final model. **(F)** External validation ROC curves for each of the final risk equation fitted to the Nepali data comparing febrile controls against typhoid cases.

To identify a combination of a small number of antigen/antibody isotypes that were predictive of typhoid challenge outcomes (TD or nTD) in a multivariable framework, we performed multivariable logistic regression. Many of the 35 selected antigen/antibody isotype combinations identified in the PLS regression were highly correlated with each other (Supplementary Figure S8A). Due to the intrinsic problems of model overfitting and model non-convergence, the 35 candidate antigen/antibody isotypes were further reduced prior to model fitting by removing those antigen/isotype combinations with the lowest frequency of selection (<30%) and combinations which consisted of two isotypes of the same antigen and were therefore highly correlated (correlation cut-off *rho* > 0.7; Supplementary Figure S8B and Supplementary Methods). This process resulted in 12 candidate antigen/antibody isotype combinations/features (**Figure 6A**). To identify the optimal feature combination we applied logistic regression to these 12 variables using step-wise back and forward feature selection (based on AIC statistics). This analysis resulted in a final model consisting of five antigen/antibody isotype combinations (**Table 2** and **Figure 6A** – gray squares; Model 1). Individual risk scores were calculated from the linear predictor of the logistic regression, i.e., the sum of the fold-change values multiplied by variable coefficients for each participant (**Figure 6B**). The bias inherent in internal validation was observed, with very high sensitivity and specificity for correctly assigning challenge outcome (AUC ROC = 0.955) seen when the model was fitted to the data from which it had been derived (**Figure 6C**). External validation on the Nepal dataset was conducted to obtain an objective estimate of the model performance in an endemic setting.

**Table 2:** Parameters of the four selected multivariable models.

	Coefficient	Standard error	*z*-value	*p*-value
**Model 1**		
(Intercept)	-1.8932	0.7273	-2.603	0.00924
IgM.t4398	-3.3309	1.4733	-2.261	0.02377
IgG.t0581	2.6645	1.2012	2.218	0.02655
IgM.t2002	2.3586	1.1021	2.14	0.03235
IgG.t1477	2.0183	0.779	2.591	0.00957
IgG.t1850	3.1504	1.0274	3.066	0.00217
Factor (Study T2)	-1.0858	1.0249	-1.059	0.28942
**Model 2**		
(Intercept)	-1.3805	0.6681	-2.066	0.03879
IgM.t4398	-4.1001	1.6237	-2.525	0.01157
IgG.t0581	2.1095	0.9966	2.117	0.03429
IgG.t1477	2.4312	1.0371	2.344	0.01907
IgM.t3090	2.2378	1.1818	1.893	0.05829
IgG.t1850	3.4462	1.0828	3.183	0.00146
Factor (Study T2)	-1.7175	1.0784	-1.593	0.11123
**Model 3**		
(Intercept)	-2.9124	1.0443	-2.789	0.00529
IgA.t2786	-2.4533	0.9472	-2.59	0.0096
IgG.t1477	1.957	0.9144	2.14	0.03234
StudyT2	-2.8085	1.6218	-1.732	0.08333
IgA_LPS	2.3243	0.6948	3.345	0.00082
**Model 4**		
(Intercept)	-1.9921	0.7006	-2.843	0.00446
IgA_LPS	1.5135	0.3565	4.245	2.18E-05
Factor (Study T2)	-0.8241	0.9093	-0.906	0.36478

IgM for putative membrane protein (t3090) was not selected in the model, likely due to its positive correlation with multiple variables. Since responses to t3090 were some of the strongest observed in both the Oxford validation and Nepal datasets (**Figures 6A,D**), we repeated the analysis forcing IgM t3090 to be kept in the model. This process resulted in a slightly modified antigen combination, which excluded IgM t2002 (putative *N*-acetylmuramoyl-L-alanine amidase) (**Table 2** and **Figure 6A** – black squares; Model 2) but had little effect on risk scores or ROC curve analysis in internal validation (AUC = 0.953; **Figures 6B,C**).

### Inclusion of Purified Proteins

Reactivity against purified antigens (LPS, flagellin, and Vi) was substantial in all three cohorts at the selected time points - Vi polysaccharide was not printed on the initial array, which was used for probing sera in the discovery set (Supplementary Figure S9). IgA responses to flagellin and LPS were highly correlated (*rho* = 0.625) with each other and other antigens in the dataset, in particular OmpA (t1850), flagellin (t0918), flagellar hook protein E (t1743), regulatory protein (t3426) and *N*-acetylmuramoyl-L-alanine amidase (t2002) (Supplementary Figure S8B). Model selection was performed with LPS forced into the model (model 3, **Table 2**), and additionally, a model with LPS alone was assessed (Model 4, **Table 2**). When LPS was forced into the model, the final model included the additional antigens IgA.t2786 and IgG.t1477 (**Figures 6A–C**).

### Assessment in an Endemic Cohort

Using the down-selected antigen array, we probed sera samples gathered from cohorts of patients in Nepal with blood culture-confirmed typhoid fever (*n* = 100; ST), patients with confirmed non-typhoid bacteremia (*n* = 52; febrile controls, FC) and healthy controls (*n* = 50; HC), to assess the performance of antigen/antibody isotype signatures identified (**Table 1**). The resulting data were heterogeneous with no apparent clustering between the healthy controls, febrile controls and typhoid patients, although more consistent clustering was observed among different isotypes (Supplementary Figure S10). The differences between typhoid samples and healthy controls for the 12 antigen/antibody isotypes used in the multivariable analysis were less pronounced in the Nepal cohort. For some antigens, the values for febrile controls were decreased when compared with healthy controls. The most distinct responses were observed for IgM.t3090 and IgA/IgG LPS (**Figure 6D**). This observation was further reflected in the risk score analysis, with moderate differences between the febrile controls and typhoid cases (**Figure 6E**).

Model 1 with 5 antigens and model 2 with t3090 forced into the model showed moderate discrimination between blood culture positive typhoid participants and febrile controls, while models containing LPS or LPS alone performed better (**Figure 6F**). A comparison of AUCs by DeLong’s test for two correlated ROC curves, showed significant differences between the performance of each of model 2, model 3 and model 4, with model 1 (*p* = 0.017, *p* = 8.8 × 10^-6^, and *p* = 6.2 × 10^-6^, respectively; **Table 3**).

**Table 3:** Comparison of receiver operator characteristic (ROC) curves in Nepal data.

**ROC model 1**	**ROC model 2**	***p*-value^1^**
0.6706	0.715	1.77E-02
**ROC model 1**	**ROC model 3**	***p*-value**
0.6706	0.8736	8.83E-06
**ROC model 1**	**ROC model 4**	***p*-value**
0.6706	0.8805	6.29E-06

^1^*DeLong’s test for two correlating ROC curves*.

## Discussion

Here we have detailed the entire breadth of the humoral response during acute typhoid fever using a comprehensive *S.* Typhi proteome array. Using samples collected in human challenge studies and an endemic setting enabled systematic identification and validation of panels of candidate diagnostic antigen/antibody isotype signatures for typhoid fever. Putative serodiagnostic antigens identified include components of the bacterial cell surface and proteins targeted toward host cell attack (HlyE) and invasion (SipC).

Successful application of antigen microarray technology to identification of candidate diagnostic or vaccine targets has been used for multiple pathogens (Liang et al., 2011a,b; Kalantari-Dehaghi et al., 2012; Liang and Felgner, 2015), but this technology has not yet been widely applied to *S.* Typhi (Bumann, 2014). A key reason may be the challenge in obtaining samples from human patients with confirmed typhoid fever in a high-incidence setting. Furthermore, interpreting the significance of seroreactivity in samples collected in an endemic setting is problematic as previous exposure or subclinical infection by typhoidal or non-typhoidal *Salmonella* is difficult to exclude. Our approach was to use samples collected during human challenge studies, thus developing a ‘cleaner’ route for antigen discovery, effectively removing the background cross-reactive antibody response (Liang et al., 2013; Davies et al., 2016). An assessment of the challenge study samples highlighted the marked heterogeneity observed in humoral responses occurring during typhoid fever and showed negligible responses in those not succumbing to overt clinical infection, despite exposure. Explanations for response heterogeneity include the complex host-pathogen interactions governed by bacterial subversion and immunomodulation, bacterial burden and variability in the magnitude of the host immune response (Wain et al., 1998; Dougan and Baker, 2014; Waddington et al., 2014).

We aimed to address variability in serological response by seeking a signature consisting of multiple antigens/antibody isotypes, rather than relying solely on a single antigen. This approach was in-keeping with the previous work by Liang et al. (2013) who identified a signature composed of either IgM or IgG responses against 10 antigens performed better than those containing fewer antigens. We applied various computational methods for selecting optimal combinations of antigen/isotype pairs as diagnostic markers. Several of the identified antigens corroborate previous findings while others are novel targets. The IgA and IgG responses to HlyE (t1477) were identified in previous typhoid microarray studies for distinguishing typhoid from controls and other febrile infections including NTS (Liang et al., 2013; Charles et al., 2014; Davies et al., 2016). Furthermore, diagnostic HlyE responses have been further validated by other laboratory techniques including ELISA (Charles et al., 2010, 2014), and dot-blot immunostrip probing (Liang et al., 2013; Davies et al., 2016). We additionally identified IgA and IgG responses to OmpA (t1850) as seroreactive in our CHIM participants. IgG responses to OmpA were classed by Liang et al. (2013) as cross-reactive, and were also identified in both acute and convalescent sera samples using an immunoproteomic screening assay in Bangladesh (Charles et al., 2014). Other *Salmonella* and Gram-negative bacteria express OmpA; therefore this antigen may not be useful in settings where background exposure is common. In the non-endemic challenge population the OmpA response was a useful discriminator for infection. Of note, comparative IgM/IgA responses to *S.* Typhi-specific OMP form the basis of the Typhidot-M test, which has been shown to distinguish typhoid infection in febrile children in Malaysia (Choo et al., 1999), but performs less well in other settings (Naheed et al., 2008; Fadeel et al., 2011; Keddy et al., 2011; Thriemer et al., 2013; Islam et al., 2016).

Flagellin has long been recognized as a potential diagnostic antigen (Calderon et al., 1986; Sadallah et al., 1990), and is a component of the Widal test (Parry et al., 2011). We detected responses to flagellin [both as the IVTT expressed antigen (t0918) and in purified form on the array] by all three isotypes, suggesting flagellin as a useful serodiagnostic classifier in the non-endemic discovery and validation cohorts. While flagellin was selected through machine learning, it was not a component of any identified signatures and correlated with responses against O-antigen. Responses against flagellin appeared to be short-lived after infection, and were not observed in all diagnosed individuals. This apparent difference in systemic exposure may account for the failure of many flagellin-based PCR assays to improve on the sensitivity of current diagnostic methods when tested with clinical samples (Tennant et al., 2015; Darton et al., 2017). Similarly, IgG and IgM responses to flagellar hook protein E (FlgE, t1743) were also significantly more reactive in TD participants in the CHIM. This structure is closely related to flagellin (Homma et al., 1990), and is likewise probably similarly cross-reactive when seen (Liang et al., 2013).

Two IgM responses to entirely novel antigen candidates were identified as being serodiagnostic in the challenge participants. These included a putative *N*-acetylmuramoyl-L-alanine amidase (t2002, STY0927), which is involved in the catabolism of peptidoglycans and has previously been associated with the invasion and intracellular survival of *Salmonella* Typhimurium (Folkesson et al., 2005). Also identified was an uncharacterized hypothetical protein, t2295. While wide ranging cross-reactivity was observed with the IgM responses, reactivity to these two antigens performed well even in the machine learning selection, with IgM against t2002 taken forward into one of the diagnostic signatures (Model 1). Multivariable analysis identified two further previously unknown antigens as part of the signature determined in Model 2. These included IgM responses to YjeN (t4398), a previously uncharacterized protein, and IgG responses to a glycerol-3-phosphate transporter (t0581, STY2512).

During machine learning analysis of the CHIM validation dataset, additional IgM responses to t3090 (a putative membrane protein) were identified as demonstrating diagnostic merit; these responses were also markedly increased in the Nepal dataset. This result was unpredicted; as previous exposure to *Salmonella* antigens would indicate that IgM responses might be less likely to predominate in an endemic setting. Therefore, we formed an additional multivariable model, forcing selection of IgM.t3090 (Model 2). Inclusion of this combination into the model altered signature composition slightly (with removal of IgM.t2002) but interestingly resulted in a significantly improved test performance when applied to the Nepal dataset (higher AUC ROC, 0.67 vs. 0.72, *p* = 0.017).

We additionally sought to evaluate how LPS and flagellin performed in combination with antigen/antibody isotype combinations selected, although responses to both antigens are known to correlate. Here, as the responses were more consistent in challenge study participants, only *S.* Typhi-specific LPS was chosen for inclusion into multivariable modeling. We found LPS correlated with many of the other antigens selected in our analyses and thus formed two models: with LPS forced into the model (Model 3) and LPS alone (Model 4). The signature suggested by model 3 also included IgG responses to HlyE and IgA responses to cell invasion protein SipC (t2786). SipC, together with SipB, forms the tip of the type-3 secretion system (T3SS) encoded by SPI-1, and is involved in bacterial pathogenesis and macrophage apoptosis, although is not specific to *S.* Typhi (Nichols and Casanova, 2010; Kaur and Jain, 2012).

While all four signatures identified performed well when reapplied to the CHIM datasets, final validation was performed using samples gathered from a typhoid-endemic setting. Overall, the reactivity in samples from the healthy control participants was high, possibly reflecting environmental exposure of the local population to *Salmonella* and other Gram-negative bacteria (Pulickal et al., 2009). Application of the multivariable models not containing LPS (Models 1 and 2) to the Nepal cohort produced a moderate ability to discriminate febrile typhoid fever patients from those with other causes of bacteremia, with the signature containing IgM.t3090 performing significantly better. However, the two LPS-containing signatures performed significantly better again in this dataset. This is a similar finding to that by Davies et al. (2016) in which the final selection of serodiagnostic antigens in a Nigerian pediatric cohort included LPS and HlyE, and IgA responses to LPS were mooted as being a useful indicator of recent infection, albeit they were cross-reactive with NTS sera.

In summary, these data offer an invaluable and unprecedented insight into the dynamics of serological responses to acute typhoid fever. Given the wide variety of settings in which typhoid is still endemic, a signature composed of multiple antigens remains likely to be the most universally useful approach to the serodiagnosis of acute typhoid fever, both in endemic settings and travelers returning from these settings. Specifically, serodiagnostic responses in a new assay should include IgA to *S.* Typhi LPS and IgG to HlyE, while several other novel combinations merit assessment in further studies.

## Materials and Methods

### Typhoid Controlled Human Infection Models (CHIM)

Human challenge with *S*. Typhi was performed as previously described (Darton et al., 2014, 2016; Waddington et al., 2014). In brief, health adult male or female volunteers between 18 and 60 years of age were challenged with a single oral dose of 10^3-4^ CFU *S*. Typhi (Quailes strain). Clinical data and samples were collected prior to challenge (day 0 or ‘baseline’) and at least daily for 14 days thereafter. In participants developing persistent fever (oral temperature ≥38°C for ≥12 h) and/or with evidence of bacteremia (Gram-negative bacilli), a typhoid diagnosis (TD) was made and antimicrobial treatment was started (first-line: ciprofloxacin 500 mg twice-daily for 14 days; **Figures 1A,B**). Diagnosed participants were seen at time points after diagnosis to ensure resolution of clinical symptoms and for collection of further sample material. All remaining participants not diagnosed with typhoid by day 14 (nTD) were also commenced on antimicrobial treatment. Further follow-up visits into convalescence were performed 28, 60, and 90 days after challenge and thereafter.

Two independent challenge cohorts were used in the present study. The first study (discovery cohort; *n* = 41) consisted of samples derived from a dose-escalation study performed in 2011 (**Figure 1A**) (Waddington et al., 2014). Serum samples were collected 0 (pre-challenge baseline), 4, 7, 10, 14, 28, 60, and 90 days after *S.* Typhi ingestion in all participants, and at two additional time points 48 h (TD48 h) and 96 h (TD96 h) after TD (also referred to as ‘TD,’ *n* = 20).

The second study (validation cohort; *n* = 30) consisted of samples collected from placebo-control arm participants taking part in a randomized, double-blind, placebo-controlled vaccine efficacy trial (Darton et al., 2016). Enrolment criteria and endpoints in this study were identical to those in the initial-dose escalation study; enrolment was completed between November 2011 and June 2012. Challenge and follow-up was performed as described above (**Figure 1B** and **Table 1**); sera samples were collected at days 0, 5, 10, 14, 28, 60, and 90 from all participants and TD48 h and TD96 h in participants diagnosed with typhoid.

### Endemic Cohort

To validate the serodiagnostic signatures in a relevant patient cohort, serum samples were gathered from three study cohorts at Patan Hospital or the Civil Hospital both located in the Lalitpur Sub-Metropolitan City area of Kathmandu Valley in Nepal. Firstly, plasma samples were collected from febrile patients presenting to hospital and diagnosed with blood culture-confirmed *S.* Typhi infection (*n* = 100; **Figure 1C** and **Table 1**). Samples were collected from patients enrolled into a parent treatment comparison study (Arjyal et al., 2016), or if ineligible, into a diagnostics sub-study; samples from healthy control volunteers were also collected as part of this sub-study. Finally, plasma samples were collected from patients presenting with fever and a laboratory confirmed of non-typhoid bacteremia (*n* = 52).

### Ethics Statement

All trials were conducted in accordance with the relevant clinical trial protocols, the principles of the Declaration of Helsinki, and the International Conference on Harmonization (ICH) Good Clinical Practice standards. Ethical approval for the Oxford CHIM studies was provided by the United Kingdom National Research Ethics Service (Oxford Research Ethics Committee A, 10/H0604/53 and 11/SC/0302) (Waddington et al., 2014; Darton et al., 2016). Both studies were performed by the University of Oxford at the Centre for Clinical Vaccinology and Tropical Medicine, Oxford, and monitored by the Clinical Trial Research Governance department of the University of Oxford.

The parent treatment trial and diagnostics sub-study performed in Nepal were given ethical approval by the Oxford Tropical Ethics Committee (OxTREC, ref. 38–11) and the Nepal Health Research Council (ref. 03NP). The trial was performed by the Oxford University Clinical Research Unit, Nepal supported and monitored by OUCRU, Vietnam. All study participants (or their parents if aged under 18 years in Nepal) provided written informed consent prior to enrolment. Illiterate signatories in Nepal, were read details of the consent form in the presence of a literate witness, who could attest to the accurate reading of the consent and agreement of the signatory.

### Sample Collection

Venous blood was collected from participants, and for the purposes described here, sera (Oxford studies) or plasma (Nepal cohort) was separated by centrifugation from clotted blood and stored at -70°C prior to separation and shipment for assays.

### Array Design

A full array of 4445 target *S*. Typhi antigens was used to assess sera from the discovery set. Antigen targets were expressed using a coupled *in vitro* transcription and translation (IVTT) system, *E. coli* based cell-free Rapid Translation System (RTS) 100 High Yield Kit (5 Prime). Approximately 1 nL of unpurified IVTT reactions were spotted onto nitrocellulose coated Oncyte Avid Slides (Grace Bio-Labs) using an OmniGrid Accent microarray printer (Digilab). Each array also contained 192 positive control spots (human IgG, IgM, IgA and anti-human IgG, IgM, IgA) and 227 negative IVTT control spots, consisting of spotted IVTT reactions with no *S*. Typhi plasmid added. A down-selected array was designed containing 223 *S*. Typhi, 6 dengue virus, 11 *P. falciparum* antigens, *S*. Typhi Vi-polysaccharide (‘Vaccine’; Sanofi Pasteur, Maidenhead, United Kingdom) and purified *S*. Typhi flagellin (‘H’; prepared by isolation from *S.* Typhi Quailes strain and purification at the University of Maryland School of Medicine) and lipopolysaccharide (LPS or ‘O,’ *S*. Typhosa L2387; Sigma–Aldrich, Dorset, United Kingdom) (Supplementary Table S2). The down-selected array was probed in duplicate for each participant sample.

### Sample Probing

Serum samples were diluted 1:100 in a 3 mg/mL *E. coli* DH5α lysate solution in protein arraying buffer and incubated at room temperature for 30 min. Chips, FAST Slide Holders and FAST Slide Incubation Chambers were assembled and nitrocellulose pads were hydrated using 100 μL blocking buffer for 30 min at room temperature with rocking. Blocking buffer was removed, pre-incubated samples were added and chips were incubated overnight at 4°C with agitation. The following day, chips were washed three times with 1x TBS-0.05% Tween 20, followed by incubation with biotin-conjugated anti-human secondary antibodies against the target antibody isotype (IgG, IgA or IgM; Sigma–Aldrich) diluted 1:1000 in blocking buffer for 1 h at room temperature with agitation. Chips were washed three times with 1x TBS-0.05% Tween 20, followed by incubation with streptavidin-conjugated SureLight P-3 (Columbia Biosciences) at room temperature protected from light with agitation. Chips were washed three times with 1X TBS-0.05% Tween 20, three times with 1X TBS, and once with water. Chips were air dried by centrifugation at 500 ×*g* for 10 min, stored in a light -proof desiccator for >2 h before scanning.

### Raw Data Processing

After probing, arrays were scanned using a GenePix 4300 scanner to acquire fluorescent intensity (FI) values for each spot on the array. Raw intensity values were determined by subtracting background FI (immediate surrounding area of a given spot) from foreground FI (spot intensity).

### Data Normalization

The raw signal was normalized by dividing the IVTT protein spot intensity by the sample specific median of the IVTT control spots printed throughout the chip and taking the base-2 logarithm of the ratio. The normalized data provide a relative measure of the specific antibody binding to the non-specific antibody binding to the IVTT control spots. With the normalized data, a value of 0.0 means the intensity is no different than background and a value of 1.0 indicates a doubling with respect to background.

### Initial Data Analysis

Overall reactivity of discovery set sera collected up to day 28 after challenge was visualized using the median raw FI of each antibody isotype (IgA, IgG, and IgM) to each antigen assayed (Supplementary Figure S1). Setting an arbitrary cut-off of 5,000 mfi, some reactivity was seen in IgA responses (to 6 antigens), while a broader range of reactivity was seen by IgM and IgG isotypes (both to 26 antigens). To compare evolution of the antibody responses from the period of *S.* Typhi ingestion into convalescence (day 28) between TD participants and those not diagnosed after challenge (also referred to as ‘nTD’), we determined the number of reactive antigens (fold-change over baseline threshold of >1.5 in >2 participants) at each time point (mean number of reactive antigens/participant; **Figure 2A**).

### Selection of Seroreactive Antigens

The reactivity of each antigen/antibody isotype combination for each participant was assessed at the day 14 time point in all participants plus the TD96 h time point in TD participants, according to the outcome after challenge. The TD96 h time point was included as, although challenge study participants were treated at the earliest opportunity after the study definitions for diagnosis had been reached, the WHO clinical case definition of typhoid requires at least 3 days of symptomatic/febrile illness (World Health Organisation, 2003). Classification as to whether each antigen was reactive or not required a 1.5-fold increase in normalized FI over individual baseline measurement. Antigen/antibody isotype combinations were selected for further investigation if reactivity was seen in 3 or more TD participants at either time point.

To assess reactivity in the validation set of those target antigens and antibody isotypes identified in the discovery set, we compared the log_2_ fold-change in FI reactivity between the TD96 h or day 14 time point and corresponding individual baseline values.

### Feature Selection Using Machine Learning Algorithms

Fold-change values (log_2_ over baseline) were created for all participants in the Oxford datasets. As the validation set was probed in duplicate, the mean FI value for each pair was used unless one spot was not above background in which case a single value was used; to avoid artificially increasing fold-change values, we set the time point log2 fold-change to zero as soon as one value was below the lower limit of detection. To select features for building a multivariable model, we combined the discovery and validation set into one large data matrix (superset). To assess algorithm performance, we created 500 training and test set partitions from the superset using random sampling with replacement (bootstrapping). For each training iteration we used internal feature importance measures to rank each feature and selected the top 20 features for further assessment. In a stepwise approach, we re-trained the training set on the top 2 to top 20 features and recorded the cross-validation accuracy. Where the accuracy was highest, these features were taken forward as classifier and used to predict the test set and balanced accuracy and AUROC values recorded as performance measures (see Supplementary Methods).

### Statistical Analysis

Statistical analysis was performed in R version 3.3.1 (2016-06-21). Differences in normalized log_2_ FI between baseline (pre-challenge) values and subsequent time point values were determined by paired *t*-tests. To identify a combination of a small number of antigen/antibody isotypes which were most predictive of typhoid challenge outcomes (TD or nTD) in a multivariable framework, we performed subset selection of multivariable logistic regression. The outcome in the model was the binary classification of typhoid diagnosis (TD = 1; nTD = 0) and the independent variables were the antigens and an indicator variable to account for the batch effect of having data from two different challenge studies (study = T1/T2).

Many of the 35 candidate antigen/antibody isotype combinations identified in the PLS machine learning approach were highly correlated with each other therefore unable to be included in a multivariable model together. The 35 candidate antigen/antibody isotypes were reduced prior to model fitting by removing those antigen/isotype combinations with the lowest frequency of selection (<30%) and those combinations which consisted of two isotypes of the same antigen and were therefore highly correlated (correlation cut-off *rho* > 0.7). This reduction in variables was conducted to overcome two potential problems: model overfitting and model non-convergence. Model overfitting occurs when fitting models with a large number of predictor variables to a relatively small dataset and results in models which do not perform well when validated on external datasets. Model non-convergence occurs when coefficients and their standard errors cannot be computed, or are exceedingly large, due to multicollinearity in the data.

The final list of candidate variable included 12 candidate antigen/antibody isotype variables. Variable subset selection using logistic regression was performed on these 12 variables using back and forward step-wise feature selection with the ‘stepAIC’ function in R ‘MASS’ package (Venables and Ripley, 2002), to select the optimal combination. Non-significant variables were excluded from the model resulting in a final model consisting of a five significant antigen/antibody isotype combination. Individual risk scores were calculated from the linear predictor (risk equation) of the logistic regression, i.e., the sum of the fold-change values multiplied by variable coefficients for each participant. Risk equations were applied to the Nepal dataset as an external validation, and the AUC ROC computed.

## Author Contributions

TD, AP, CB, and SB designed the study and analysis. TD, SD, AK, AA, MC, CJ, JA, and CB performed the studies and collected sample material. TD, CB, DM, AR, and MV carried out the analyses. AR, KT, JP, CH, AT, AS, TL, CW, and DM performed the assays. TD and CB wrote the manuscript. TD and AP acquired the funding. All authors provided input to and approved the manuscript.

## Conflict of Interest Statement

The authors declare that the research was conducted in the absence of any commercial or financial relationships that could be construed as a potential conflict of interest.
