# Supplementary material for: Identification of Novel Serodiagnostic Signatures of Typhoid Fever Using a Salmonella Proteome Array
Source: Front Microbiol. 2017 Sep 19;8:1794. doi: 10.3389/fmicb.2017.01794 (PMC5609549; doi:10.3389/fmicb.2017.01794)
Supplement: Supplementary file 1 [file Data_Sheet_1.docx]

Supplementary Material

Identification of novel serodiagnostic signatures of typhoid fever using a Salmonella proteome array

Thomas C. Darton^1,2,3*^, Stephen Baker^2^, Arlo Randall^4^, Sabina Dongol^5^, Abhilasha Karkey^5^, Merryn Voysey^1,6^, Michael J. Carter^1^, Claire Jones^1^, Krista Trappl^4^, Jozelyn Pablo^4^, Chris Hung^4^, Andy Teng^4^, Adam Shandling^4^, Tim Le^4^, Cassidy Walker^4^, Douglas Molina^4^, Jason Andrews^7^, Amit Arjyal^6^, Buddha Basnyat^6^, Andrew J. Pollard^1†^, Christoph J. Blohmke^1†^

*** Correspondence:** Thomas C Darton: Thomas.darton@paediatrics.ox.ac.uk

# Supplementary Methods

## Antigen Down-Selection (16-pad array)

The contents of the down selected array were chosen primarily based on results observed in three human studies with the full proteome array: a vaccine study (IgG; T2) (Darton et al., 2016), a dose-escalation challenge study (IgG, IgA, IgM; Discovery Set; T1)(Waddington et al., 2014), and a clinical study performed in Nepal (IgG, IgA; pilot on Nepali cohort). For the T1 and T2 studies, antigens were selected for higher responses following vaccination or challenge. For the Nepalese clinical study, antigens were selected for differential responses (lower or higher) when comparing the control and *S.* Typhi positive samples. A small number of additional antigens described as differentially reactive in prior studies were also included in the selection. In addition, purified *S*. Typhi flagellin and LPS antigens as well as Vi polysaccharide was included. Finally, 6 dengue virus and 11 *P.* *falciparum* were identified and included in the array (**Table S1**).

## Array data processing Validation Set

Because the data of the validation set was generated in duplicates, we averaged log2-normalized intensities of both replicates. The lower limit of detection (LLD) of log2-normalized intensities was -2 and single intensity values were used when one replicate was below the LLD and the LLD in case both values were not measurable.

## Data QC for machine learning

In order to achieve reasonable training and test set sizes in the prediction analysis we combined the discovery and validation cohort including only antigen features common to both arrays (superset matrix). To ensure maximum antigen responses close to the period of acute disease and comparable between those diagnosed and those who stayed well following challenge, we included samples collected at day 14 after challenge. In participants diagnosed with acute typhoid fever after day 10 after challenge we used the time point TD+96hrs. This resulted in 25 participants who stayed well following challenge and 39 participants who were diagnosed. Antigens which had a value set to the LLD (log_2_Int = -2) due to technical artefacts in more than 5 participants were removed (5 spots: IgG.t3941, IgM.t3941, IgA.t3941, IgM.t3001, IgM.t3355). Remaining antigens with signals at the LLD in either baseline or post-challenge sample were set to a log_2_ fold-change of 0 to avoid the fold-change value being artificially confounded. The remaining 715 features (isotype-antigen combinations) were assessed for expression quality using unsupervised PCA and hierarchical clustering analyses.

## Model development

We used the caret() package in the R statistical environment (R Core Team, 2017), in order to test several prediction algorithms and identify isotype antigen combinations with the best predictive power.

### Partitions

In order to avoid effects non-representative of a random cohort, we partitioned our superset matrix into 500 random, bootstrapped test and training sets using the createDataPartition() function in the Caret package (Kuhn, 2012). These partitions were run each in the entire algorithm below.

### Feature ranking and selection

Several different machine learning algorithms were used in the model development approach. Initially, for each training/test set partition, each model was trained using all 715 antigen-isotype features and a leave-one-out-cross-validation (LOOCV) approach. Features were then ranked by importance for each model using the varImp() function. Using this ranking, the top 20 features were selected to retrain each algorithm iteratively using the top 2 features and adding one feature/iteration subsequently. For each run the misclassification rate of the LOOCV was recorded. The features (classifier) that resulted in the lowest misclassification rate were put forward to the training and testing step.

### Re-training and testing

The training and test set was subsetted based on the classifier identified in each iteration. Each model was retrained using the smaller training set and then applied to predict the test set. Balanced accuracies and area under the ROC curve (AUROC) were recorded for all 500 random partitions.

### Multivariate analysis to identify a final model

Out of 35 antigens identified in the machine learning approach, we removed the antigens which were highly correlated with each other (Spearman’s *rho* > 0.7). Out of two correlating features, the one with the lower effect size was removed. This approach resulted in 12 unique isotype antigen features to be selected and taken forward into the multivariate analysis.

# Supplementary Figures and Tables

## Supplementary Figures

**Figure S1: Median raw intensity of antibody response signals to 4445 antigens in samples collected in a human challenge.** Intensity was calculated across all time points (D0 – D28) and groups (TD and nTD). Fluorescent intensities above 5000 are highlighted red

**Figure S2:** Longitudinal profiles of antigen responses to remaining 3 (IgA), 4 (IgG) and 12 (IgM) antigens identified in the discovery set. A-C) Participants diagnosed with typhoid fever (TD). D-F) Participants who stayed well during the challenge period (nTD).

.

**Figure S3: Selection of four representative antigen/antibody isotype responses in participants who stayed well following challenge with oral *Salmonella* Typhi (nTD).** A) IgA responses. B) IgG responses. C) IgM responses. D) Responses to purified flagellin (0.1ug) and lipopolysaccharide (LPS, 0.1ug) as additional antigens included on the array

**Figure S4.** Receiver operator characteristic curves for the antigen/antibody isotype combinations with respect to their ability for fold-change increases in FI between baseline and day 14 to predict challenge outcome (study diagnosis of typhoid or not) in both discovery and validation sets. AUC 95% confidence intervals calculated by the method of De Long *et al* (14). Black curves, AUC ROC 95% LCL>0.5; grey curves, 95% LCL≤0.5.

**Figure S5: PCA of fold-change values in all three cohorts based on antigen/isotype features common to all three studies.** In the Oxford challenge studies, data from day 14 in those who stayed well and day 14 or TD96hrs (whatever was later) in those who developed acute typhoid fever was used. CTRL, control; FC, febrile control; ST, *Salmonella* Typhi. T1, first “discovery” challenge study; T2, second “validation” challenge study; Nep, Nepal “validation” study.

**Figure S6: Classifier sizes.** For each model (rows) the prediction performance (ROC AUC) was plotted across all 500 iterations against classifier size as A) binned representation with low (2-6 classifier), medium (7-13 classifiers) and high (14-20 classifiers) sizes and B) for individual sizes (2-20).

**Figure S7: Feature selection based on the linear SVM algorithm applied to the Oxford challenge datasets.** A) Frequency of features selected in each of the 500 bootstrapped data samples using the linear SVM model. B) Feature frequencies for features selected as part of a classifier in >10% of the 500 bootstrapped data samples in the linear SVM. Frequencies are split by classifier size. The last column represents the overall frequency across all 500 iterations.

**Figure S8: Correlation matrices of features extracted from the PLS model before (A) and after (B) removal of highly correlating features *(rho*>0.7)*.***

**Figure S9: Purified antigens in the (A) discovery set (flagellin and LPS only), (B) the validation set and the Nepal cohort (C).** Data is shown as log2 fold-changes at day 14 or TD96 compared to paired baselines (A&B) or compared to the median of the healthy control population (C). Flg: flagellin; LPS: lipopolysaccharide; Vi: Vi polysaccharide.

**Figure S10: Heatmap representation of Nepal protein microarray data.** Cond, condition. Iso, antibody isotype. HC, healthy control; FC, febrile control; ST, *Salmonella* Typhi.

**Table S1: Antigens selected as seroreactive in the discovery cohort.**

| **Number** | **Antigen** | **Isotype** | **Antigen Names** |
| --- | --- | --- | --- |
| 1 | t0918 | IgA | Flagellin |
| 2 | t1743 | IgA | flagellar hook protein FlgE |
| 3 | t3426 | IgA | regulatory protein |
| 4 | t1477 | IgA | hemolysin E |
| 5 | t1850 | IgA | outer membrane protein A |
| 6 | t0180 | IgA | hypothetical protein t0180 |
| 7 | t4239 | IgA | hypothetical protein t4239 |
|  |  |  |  |
| 1 | t0918 | IgG | flagellin |
| 2 | t1477 | IgG | hemolysin E |
| 3 | t1850 | IgG | outer membrane protein A |
| 4 | t2919 | IgG | 2-acylglycerophosphoethanolamine acyl transferase (aas) |
| 5 | t1300 | IgG | hypothetical protein t1300 |
| 6 | t0101 | IgG | IS element transposase |
| 7 | t1485 | IgG | methyl viologen resistance protein SmvA |
| 8 | t0596 | IgG | phosphotransfer intermediate protein in two-component regulatory system with RcsBC |
|  |  |  |  |
| 1 | t0918 | IgM | flagellin |
| 2 | t1743 | IgM | flagellar hook protein FlgE |
| 3 | t3426 | IgM | regulatory protein |
| 4 | t2421 | IgM | cytochrome o ubiquinol oxidase C subunit (cyoE) |
| 5 | t1506 | IgM | putative aldehyde dehydrogenase |
| 6 | t1590 | IgM | putative ATP-binding protein |
| 7 | t3828 | IgM | autotransporter |
| 8 | t3709 | IgM | heat shock protein IbpA |
| 9 | t2295 | IgM | hypothetical protein t2295 |
| 10 | t2656 | IgM | hypothetical protein t2656 |
| 11 | t0371 | IgM | lipoprotein |
| 12 | t1224 | IgM | lipoprotein |
| 13 | t3199 | IgM | lipoprotein NlpI |
| 14 | t2002 | IgM | N-acetylmuramoyl-L-alanine amidase |
| 15 | t4312 | IgM | regulatory protein |
| 16 | t4164 | IgM | type I secretion protein |

## Supplementary Tables

**Table S2: Antigens contained on the downselected 16-PAD array**

| **Pathogen** | **Spot.Type** | **Gene.ID** |  | **Pathogen** | **Spot.Type** | **Gene.ID** |
| --- | --- | --- | --- | --- | --- | --- |
| Dengue | IVTT.PROTEIN | Envelope.ST1 |  | S. Typhi | IVTT.PROTEIN | t0918 |
| Dengue | IVTT.PROTEIN | Envelope.ST3 |  | S. Typhi | IVTT.PROTEIN | t3825 |
| Dengue | IVTT.PROTEIN | Envelope.ST4 |  | S. Typhi | IVTT.PROTEIN | t1743 |
| Dengue | IVTT.PROTEIN | NS1.ST1 |  | S. Typhi | IVTT.PROTEIN | t2315 |
| Dengue | IVTT.PROTEIN | NS1.ST3 |  | S. Typhi | IVTT.PROTEIN | t3981 |
| Dengue | IVTT.PROTEIN | NS1.ST4 |  | S. Typhi | IVTT.PROTEIN | t0391 |
| P. falciparum | IVTT.PROTEIN | PF3D7_0206800 |  | S. Typhi | IVTT.PROTEIN | t0518 |
| P. falciparum | IVTT.PROTEIN | PF3D7_0206900.1.1o2 |  | S. Typhi | IVTT.PROTEIN | t1111 |
| P. falciparum | IVTT.PROTEIN | PF3D7_0207000.e1 |  | S. Typhi | IVTT.PROTEIN | t2006 |
| P. falciparum | IVTT.PROTEIN | PF3D7_0220000.e2s1 |  | S. Typhi | IVTT.PROTEIN | t2305 |
| P. falciparum | IVTT.PROTEIN | PF3D7_0304600 |  | S. Typhi | IVTT.PROTEIN | t2351 |
| P. falciparum | IVTT.PROTEIN | PF3D7_0424100.2o2 |  | S. Typhi | IVTT.PROTEIN | t2382 |
| P. falciparum | IVTT.PROTEIN | PF3D7_0731500.s2 |  | S. Typhi | IVTT.PROTEIN | t3426 |
| P. falciparum | IVTT.PROTEIN | PF3D7_0930300.s2 |  | S. Typhi | IVTT.PROTEIN | t3709 |
| P. falciparum | IVTT.PROTEIN | PF3D7_1035300.iso1.exon1.amp1 | | S. Typhi | IVTT.PROTEIN | t3806 |
| P. falciparum | IVTT.PROTEIN | PF3D7_1036400.1o2 |  | S. Typhi | IVTT.PROTEIN | t3851 |
| P. falciparum | IVTT.PROTEIN | PF3D7_1133400 |  | S. Typhi | IVTT.PROTEIN | t4253 |
| S. Typhi | Vaccine | Vi 0.4 mg/ml |  | S. Typhi | IVTT.PROTEIN | t0180 |
| S. Typhi | Vaccine | Vi 10 mg/ml |  | S. Typhi | IVTT.PROTEIN | t0248 |
| S. Typhi | Vaccine | Vi 20 mg/ml |  | S. Typhi | IVTT.PROTEIN | t0500 |
| S. Typhi | Vaccine | Vi 50 mg/ml |  | S. Typhi | IVTT.PROTEIN | t0667 |
| S. Typhi | PURIFIED.PROTEIN | H-antigen 0.01 |  | S. Typhi | IVTT.PROTEIN | t0787 |
| S. Typhi | PURIFIED.PROTEIN | H-antigen 0.03 |  | S. Typhi | IVTT.PROTEIN | t1016 |
| S. Typhi | PURIFIED.PROTEIN | H-antigen 0.1 |  | S. Typhi | IVTT.PROTEIN | t1245 |
| S. Typhi | PURIFIED.PROTEIN | S. Typhi LPS 0.01 |  | S. Typhi | IVTT.PROTEIN | t1267 |
| **Pathogen** | **Spot.Type** | **Gene.ID** |  | **Pathogen** | **Spot.Type** | **Gene.ID** |
| S. Typhi | PURIFIED.PROTEIN | S. Typhi LPS 0.03 |  | S. Typhi | IVTT.PROTEIN | t1459 |
| S. Typhi | PURIFIED.PROTEIN | S. Typhi LPS 0.1 |  | S. Typhi | IVTT.PROTEIN | t1579 |
| S. Typhi | IVTT.PROTEIN | t1477 |  | S. Typhi | IVTT.PROTEIN | t1714 |
| S. Typhi | IVTT.PROTEIN | t1850 |  | S. Typhi | IVTT.PROTEIN | t1852 |
| S. Typhi | IVTT.PROTEIN | t1859 |  | S. Typhi | IVTT.PROTEIN | t0203 |
| S. Typhi | IVTT.PROTEIN | t1978 |  | S. Typhi | IVTT.PROTEIN | t0224 |
| S. Typhi | IVTT.PROTEIN | t2002 |  | S. Typhi | IVTT.PROTEIN | t0236 |
| S. Typhi | IVTT.PROTEIN | t2095 |  | S. Typhi | IVTT.PROTEIN | t0247 |
| S. Typhi | IVTT.PROTEIN | t2274 |  | S. Typhi | IVTT.PROTEIN | t0264 |
| S. Typhi | IVTT.PROTEIN | t2427 |  | S. Typhi | IVTT.PROTEIN | t0325 |
| S. Typhi | IVTT.PROTEIN | t2868 |  | S. Typhi | IVTT.PROTEIN | t0331 |
| S. Typhi | IVTT.PROTEIN | t2894 |  | S. Typhi | IVTT.PROTEIN | t0346 |
| S. Typhi | IVTT.PROTEIN | t2919 |  | S. Typhi | IVTT.PROTEIN | t0371 |
| S. Typhi | IVTT.PROTEIN | t2941 |  | S. Typhi | IVTT.PROTEIN | t0377 |
| S. Typhi | IVTT.PROTEIN | t3005 |  | S. Typhi | IVTT.PROTEIN | t0379 |
| S. Typhi | IVTT.PROTEIN | t3026 |  | S. Typhi | IVTT.PROTEIN | t0386 |
| S. Typhi | IVTT.PROTEIN | t3397 |  | S. Typhi | IVTT.PROTEIN | t0392 |
| S. Typhi | IVTT.PROTEIN | t3705 |  | S. Typhi | IVTT.PROTEIN | t0510 |
| S. Typhi | IVTT.PROTEIN | t3737 |  | S. Typhi | IVTT.PROTEIN | t0519 |
| S. Typhi | IVTT.PROTEIN | t3872 |  | S. Typhi | IVTT.PROTEIN | t0536 |
| S. Typhi | IVTT.PROTEIN | t3878 |  | S. Typhi | IVTT.PROTEIN | t0539 |
| S. Typhi | IVTT.PROTEIN | t3898 |  | S. Typhi | IVTT.PROTEIN | t0581 |
| S. Typhi | IVTT.PROTEIN | t3954 |  | S. Typhi | IVTT.PROTEIN | t0586 |
| S. Typhi | IVTT.PROTEIN | t4014 |  | S. Typhi | IVTT.PROTEIN | t0594 |
| S. Typhi | IVTT.PROTEIN | t4103 |  | S. Typhi | IVTT.PROTEIN | t0596 |
| S. Typhi | IVTT.PROTEIN | t4481 |  | S. Typhi | IVTT.PROTEIN | t0612 |
| S. Typhi | IVTT.PROTEIN | t4510 |  | S. Typhi | IVTT.PROTEIN | t0693 |
| S. Typhi | IVTT.PROTEIN | t4521 |  | S. Typhi | IVTT.PROTEIN | t0700 |
|  |  |  |  |  |  |  |
| **Pathogen** | **Spot.Type** | **Gene.ID** |  | **Pathogen** | **Spot.Type** | **Gene.ID** |
| S. Typhi | IVTT.PROTEIN | t0011 |  | S. Typhi | IVTT.PROTEIN | t0732 |
| S. Typhi | IVTT.PROTEIN | t0020 |  | S. Typhi | IVTT.PROTEIN | t0769 |
| S. Typhi | IVTT.PROTEIN | t0040 |  | S. Typhi | IVTT.PROTEIN | t0788 |
| S. Typhi | IVTT.PROTEIN | t0101 |  | S. Typhi | IVTT.PROTEIN | t0885 |
| S. Typhi | IVTT.PROTEIN | t0137 |  | S. Typhi | IVTT.PROTEIN | t0977 |
| S. Typhi | IVTT.PROTEIN | t0994 |  | S. Typhi | IVTT.PROTEIN | t1935 |
| S. Typhi | IVTT.PROTEIN | t1012 |  | S. Typhi | IVTT.PROTEIN | t1949 |
| S. Typhi | IVTT.PROTEIN | t1051 |  | S. Typhi | IVTT.PROTEIN | t2048 |
| S. Typhi | IVTT.PROTEIN | t1107 |  | S. Typhi | IVTT.PROTEIN | t2070 |
| S. Typhi | IVTT.PROTEIN | t1116 |  | S. Typhi | IVTT.PROTEIN | t2110 |
| S. Typhi | IVTT.PROTEIN | t1119 |  | S. Typhi | IVTT.PROTEIN | t2126 |
| S. Typhi | IVTT.PROTEIN | t1128 |  | S. Typhi | IVTT.PROTEIN | t2127 |
| S. Typhi | IVTT.PROTEIN | t1155 |  | S. Typhi | IVTT.PROTEIN | t2129 |
| S. Typhi | IVTT.PROTEIN | t1224 |  | S. Typhi | IVTT.PROTEIN | t2241 |
| S. Typhi | IVTT.PROTEIN | t1266 |  | S. Typhi | IVTT.PROTEIN | t2242 |
| S. Typhi | IVTT.PROTEIN | t1279 |  | S. Typhi | IVTT.PROTEIN | t2291 |
| S. Typhi | IVTT.PROTEIN | t1285 |  | S. Typhi | IVTT.PROTEIN | t2295 |
| S. Typhi | IVTT.PROTEIN | t1300 |  | S. Typhi | IVTT.PROTEIN | t2421 |
| S. Typhi | IVTT.PROTEIN | t1358 |  | S. Typhi | IVTT.PROTEIN | t2461 |
| S. Typhi | IVTT.PROTEIN | t1449 |  | S. Typhi | IVTT.PROTEIN | t2476 |
| S. Typhi | IVTT.PROTEIN | t1485 |  | S. Typhi | IVTT.PROTEIN | t2491 |
| S. Typhi | IVTT.PROTEIN | t1501 |  | S. Typhi | IVTT.PROTEIN | t2538 |
| S. Typhi | IVTT.PROTEIN | t1503 |  | S. Typhi | IVTT.PROTEIN | t2545 |
| S. Typhi | IVTT.PROTEIN | t1506 |  | S. Typhi | IVTT.PROTEIN | t2583 |
| S. Typhi | IVTT.PROTEIN | t1544 |  | S. Typhi | IVTT.PROTEIN | t2619 |
| S. Typhi | IVTT.PROTEIN | t1548 |  | S. Typhi | IVTT.PROTEIN | t2656 |
| S. Typhi | IVTT.PROTEIN | t1590 |  | S. Typhi | IVTT.PROTEIN | t2695 |
| S. Typhi | IVTT.PROTEIN | t1593 |  | S. Typhi | IVTT.PROTEIN | t2698 |
| **Pathogen** | **Spot.Type** | **Gene.ID** |  | **Pathogen** | **Spot.Type** | **Gene.ID** |
| S. Typhi | IVTT.PROTEIN | t1700 |  | S. Typhi | IVTT.PROTEIN | t2712 |
| S. Typhi | IVTT.PROTEIN | t1701 |  | S. Typhi | IVTT.PROTEIN | t2758 |
| S. Typhi | IVTT.PROTEIN | t1713 |  | S. Typhi | IVTT.PROTEIN | t2776 |
| S. Typhi | IVTT.PROTEIN | t1797 |  | S. Typhi | IVTT.PROTEIN | t2786 |
| S. Typhi | IVTT.PROTEIN | t1803 |  | S. Typhi | IVTT.PROTEIN | t2787 |
| S. Typhi | IVTT.PROTEIN | t1814 |  | S. Typhi | IVTT.PROTEIN | t2800 |
| S. Typhi | IVTT.PROTEIN | t1856 |  | S. Typhi | IVTT.PROTEIN | t2864 |
| S. Typhi | IVTT.PROTEIN | t2915 |  | S. Typhi | IVTT.PROTEIN | t3515 |
| S. Typhi | IVTT.PROTEIN | t2926 |  | S. Typhi | IVTT.PROTEIN | t3525 |
| S. Typhi | IVTT.PROTEIN | t2957 |  | S. Typhi | IVTT.PROTEIN | t3555 |
| S. Typhi | IVTT.PROTEIN | t2964 |  | S. Typhi | IVTT.PROTEIN | t3613 |
| S. Typhi | IVTT.PROTEIN | t2970 |  | S. Typhi | IVTT.PROTEIN | t3646 |
| S. Typhi | IVTT.PROTEIN | t2975 |  | S. Typhi | IVTT.PROTEIN | t3708 |
| S. Typhi | IVTT.PROTEIN | t2988 |  | S. Typhi | IVTT.PROTEIN | t3710 |
| S. Typhi | IVTT.PROTEIN | t3059 |  | S. Typhi | IVTT.PROTEIN | t3757 |
| S. Typhi | IVTT.PROTEIN | t3063 |  | S. Typhi | IVTT.PROTEIN | t3828 |
| S. Typhi | IVTT.PROTEIN | t3065 |  | S. Typhi | IVTT.PROTEIN | t3874 |
| S. Typhi | IVTT.PROTEIN | t3090 |  | S. Typhi | IVTT.PROTEIN | t3904 |
| S. Typhi | IVTT.PROTEIN | t3116 |  | S. Typhi | IVTT.PROTEIN | t3908 |
| S. Typhi | IVTT.PROTEIN | t3119 |  | S. Typhi | IVTT.PROTEIN | t3929 |
| S. Typhi | IVTT.PROTEIN | t3136 |  | S. Typhi | IVTT.PROTEIN | t3941 |
| S. Typhi | IVTT.PROTEIN | t3175 |  | S. Typhi | IVTT.PROTEIN | t3942 |
| S. Typhi | IVTT.PROTEIN | t3199 |  | S. Typhi | IVTT.PROTEIN | t3965 |
| S. Typhi | IVTT.PROTEIN | t3200 |  | S. Typhi | IVTT.PROTEIN | t4010 |
| S. Typhi | IVTT.PROTEIN | t3213 |  | S. Typhi | IVTT.PROTEIN | t4011 |
| S. Typhi | IVTT.PROTEIN | t3222 |  | S. Typhi | IVTT.PROTEIN | t4024 |
| S. Typhi | IVTT.PROTEIN | t3267 |  | S. Typhi | IVTT.PROTEIN | t4052 |
|  |  |  |  |  |  |  |
|  |  |  |  |  |  |  |
| **Pathogen** | **Spot.Type** | **Gene.ID** |  | **Pathogen** | **Spot.Type** | **Gene.ID** |
| S. Typhi | IVTT.PROTEIN | t3284 |  | S. Typhi | IVTT.PROTEIN | t4233 |
| S. Typhi | IVTT.PROTEIN | t3291 |  | S. Typhi | IVTT.PROTEIN | t4239 |
| S. Typhi | IVTT.PROTEIN | t3315 |  | S. Typhi | IVTT.PROTEIN | t4290 |
| S. Typhi | IVTT.PROTEIN | t3316 |  | S. Typhi | IVTT.PROTEIN | t4312 |
| S. Typhi | IVTT.PROTEIN | t3324 |  | S. Typhi | IVTT.PROTEIN | t4322 |
| S. Typhi | IVTT.PROTEIN | t3355 |  | S. Typhi | IVTT.PROTEIN | t4356 |
| S. Typhi | IVTT.PROTEIN | t3362 |  | S. Typhi | IVTT.PROTEIN | t4398 |
| S. Typhi | IVTT.PROTEIN | t3444 |  | S. Typhi | IVTT.PROTEIN | t4513 |
| S. Typhi | IVTT.PROTEIN | t3506 |  | S. Typhi | IVTT.PROTEIN | t4562_m2 |
| S. Typhi | IVTT.PROTEIN | t3509 |  | S. Typhi | IVTT.PROTEIN | t4600 |
| S. Typhi | IVTT.PROTEIN | t3513 |  | S. Typhi | IVTT.PROTEIN | t1649 |
| S. Typhi | IVTT.PROTEIN | t4090 |  | S. Typhi | IVTT.PROTEIN | t3210 |
| S. Typhi | IVTT.PROTEIN | t4155 |  | S. Typhi | IVTT.PROTEIN | t0429 |
| S. Typhi | IVTT.PROTEIN | t4164 |  | S. Typhi | IVTT.PROTEIN | t0294 |
| S. Typhi | IVTT.PROTEIN | t4189 |  | S. Typhi | IVTT.PROTEIN | t2569 |
| S. Typhi | IVTT.PROTEIN | t4204 |  | S. Typhi | IVTT.PROTEIN | t4166 |
| S. Typhi | IVTT.PROTEIN | t4225 |  | S. Typhi | IVTT.PROTEIN | t0058 |

**Table S3: Target antigens selected by different approaches: AUROC or partial least squares.**

| **Antibody isotype** | **ROC^1^** | **PLS^2^** | **Antigen name** |
| --- | --- | --- | --- |
| **IgA** | IgA.t1850 | IgA.t1850 | outer membrane protein A |
|  | IgA.t1477 | IgA.t1477 | hemolysin E |
|  | - | IgA.t3315 | multifunctional fatty acid oxidation complex subunit alpha |
|  | - | IgA.t3355 | hypothetical protein t3355 |
|  | - | IgA.t3284_m2_s2 | possible exported protein |
|  | - | IgA.t2786 | pathogenicity island 1 effector protein |
|  | IgA.t0918 | IgA.t0918 | flagellin |
|  | - | IgA.t3090 | hypothetical protein t3090 |
|  | IgA.t1743 | IgA.t1743 | flagellar hook protein FlgE |
| **IgG** | IgG.t1850 | IgG.t1850 | outer membrane protein A |
|  | IgG.t1477 | IgG.t1477 | hemolysin E |
|  | - | IgG.t1485 | methyl viologen resistance protein SmvA |
|  | - | IgG.t3090 | hypothetical protein t3090 |
|  | - | IgG.t2800 | virulence-associated secretory protein |
|  | - | IgG.t3119 | hypothetical protein t3119 |
|  | - | IgG.t2127 | peptidoglycan-associated outer membrane lipoprotein |
|  | - | IgG.t0581 | sn-glycerol-3-phosphate transporter |
|  | - | IgG.t2919 | 2-acylglycerophosphoethanolamine acyl transferase (aas) |
|  | - | IgG.t0918 | flagellin |
| **IgM** | - | IgM.t3090 | hypothetical protein t3090 |
|  | IgM.t2002 | IgM.t2002 | N-acetylmuramoyl-L-alanine amidase |
|  | - | IgM.t2129 | cell envelope integrity inner membrane protein TolA |
|  | IgM.t1590 | IgM.t1590 | putative ATP-binding protein |
|  | - | IgM.t4398 | hypothetical protein t4398 |
|  | - | IgM.t4290 | hypothetical protein t4290 |
|  | - | IgM.t1544_s1 | ATP-dependent helicase HrpA (hrpA) |
|  | - | IgM.t1852 | conserved hypothetical protein |
|  | - | IgM.t0371 | lipoprotein |
|  | IgM.t3709 | IgM.t3709 | heat shock protein IbpA |
|  | - | IgM.t4312 | regulatory protein |
|  | - | IgM.t1501 | hypothetical protein t1501 |
|  | IgM.t1743 | IgM.t1743 | flagellar hook protein FlgE |
|  | - | IgM.t2800 | virulence-associated secretory protein |
|  | IgM.t2295 | IgM.t2295 | hypothetical protein t2295 |
|  | IgM.t3426 | IgM.t3426 | regulatory protein |

^1^ Threshold: AUC LCL>0.5

^2^ Threshold: overall % >10%

Darton, T.C., Jones, C., Blohmke, C.J., Waddington, C.S., Zhou, L., Peters, A., et al. (2016). Using a Human Challenge Model of Infection to Measure Vaccine Efficacy: A Randomised, Controlled Trial Comparing the Typhoid Vaccines M01ZH09 with Placebo and Ty21a. *PLoS Negl Trop Dis* 10(8)**,** e0004926. doi: 10.1371/journal.pntd.0004926.

Kuhn, M. 2012. caret: Classification and Regression Training. *R package version 5.15-044.* [http://cran.r-project.org/package=caret](http://CRAN.R-project.org/package=caret) [Online]. Available: [http://cran.r-project.org/package=caret](http://CRAN.R-project.org/package=caret).

R Core Team (2017). "R: A language and environment for statistical computing", (ed.) V. R Foundation for Statistical Computing, Austria,.).

Waddington, C.S., Darton, T.C., Jones, C., Haworth, K., Peters, A., John, T., et al. (2014). An outpatient, ambulant-design, controlled human infection model using escalating doses of Salmonella Typhi challenge delivered in sodium bicarbonate solution. *Clin Infect Dis* 58(9)**,** 1230-1240. doi: 10.1093/cid/ciu078.
